# Few-shot learning for joint model in underwater acoustic target recognition

**DOI:** 10.1038/s41598-023-44641-2

**Published:** 2023-10-16

**Authors:** Shengzhao Tian, Di Bai, Junlin Zhou, Yan Fu, Duanbing Chen

**Affiliations:** 1 Big Data Research Center, University of Electronic Science and Technology of China, Chengdu, 611731 China; 2 Suining Municipal Government Services and Big Data Administration, Suining, 629018 China; 3 Chengdu Union Big Data Tech. Inc., Chengdu, 610041 China; 4 Suining Institute of Digital Economy, Suining, 629018 China

**Keywords:** Computer science, Information technology

## Abstract

In underwater acoustic target recognition, there is a lack of massive high-quality labeled samples to train robust deep neural networks, and it is difficult to collect and annotate a large amount of base class data in advance unlike the image recognition field. Therefore, conventional few-shot learning methods are difficult to apply in underwater acoustic target recognition. In this report, following advanced self-supervised learning frameworks, a learning framework for underwater acoustic target recognition model with few samples is proposed. Meanwhile, a semi-supervised fine-tuning method is proposed to improve the fine-tuning performance by mining and labeling partial unlabeled samples based on the similarity of deep features. A set of small sample datasets with different amounts of labeled data are constructed, and the performance baselines of four underwater acoustic target recognition models are established based on these datasets. Compared with the baselines, using the proposed framework effectively improves the recognition effect of four models. Especially for the joint model, the recognition accuracy has increased by 2.04% to 12.14% compared with the baselines. The model performance on only 10 percent of the labeled data can exceed that on the full dataset, effectively reducing the dependence of model on the number of labeled samples. The problem of lack of labeled samples in underwater acoustic target recognition is alleviated.

## Introduction

In recent years, many methods have attempted to establish end-to-end deep neural networks to identify underwater acoustic targets by automatically extracting deep features^1^. However, because of expensive annotation cost and inevitable annotation errors^2^, there is a lack of massive high-quality labeled samples to train robust deep neural networks in underwater acoustic target recognition^3^.

The lack of labeled samples is a common pain point faced by all deep learning models. Many studies have focused on few-shot learning (FSL)^4^, which is driven by *N*-way *K*-shot learning tasks, where *N* is the number of categories and *K* is the number of labeled samples in each category. The few-shot learning method usually solve the recognition problem of few samples categories by leveraging other classes with massive labeled samples to help the model gain some class-independent capabilities. The dataset composed of other classes is called base class dataset. Correspondingly, the dataset composed of target *N*-way *K*-shot tasks is called new class dataset. There is no overlap of categories between base class dataset and new class dataset. With the continuous development of deep learning, the researches on few-shot learning have shown a blowout development^5, 6^. Ghavidel et al.^7^ explored this kind of method in underwater acoustic target recognition and achieved satisfactory recognition performances under 5-way 1-shot and 5-way 5-shot tasks. However, in underwater acoustic target recognition, there are few categories of samples. It is difficult to collect and annotate a large amount of base class data in advance unlike the image recognition field. Therefore, such few-shot learning methods are difficult to apply in underwater acoustic target recognition.

In fact, the recording samples are sufficient in the real application scenario of underwater acoustic target recognition. Most underwater acoustic acquisition equipments can automatically collect and store underwater acoustic recording for a long time. Therefore, a common scenario is recognizing underwater acoustic targets under the setting of massive unlabeled samples and few labeled samples.

Recently, an increasing number of researches have focused on self-supervised learning of deep neural networks^8^. Most of the researches were carried out under the setting of massive unlabeled samples and few labeled samples. Specifically, massive unlabeled samples were used in self-supervised learning to enable the model to learn effective visual representations. The few labeled samples from different downstream tasks or datasets were used in supervised fine-tuning to make the model perform well on specific recognition tasks. Sohn et al.^9^ proposed a semi-supervised learning methods (FixMatch) in which weakly-augmented images were used to obtain pseudo labels and strongly-augmented images were used to predict the corresponding pseudo labels. Chen et al.^10^ proposed a simple framework for contrastive learning of visual representations (SimCLR), in which model learns effective representation by learning the differences between different samples and the consistency between the same samples enhanced by different data augmentations. Using unsupervised knowledge distillation after supervised fine-tuning further improves the performance of model on specific task (SimCLRv2)^11^. He et al.^12^ presented momentum contrast (MoCo) for unsupervised visual representation learning. A dynamic dictionary with a queue and a moving-averaged encoder was used to train the visual representation encoder by matching an encoded query to a dictionary of encoded keys using a contrastive loss. The competitive results were provided by using an MLP projection head and more data augmentations^13^. Caron et al.^14^ proposed SwAV that simultaneously clusters the data while enforcing consistency between cluster assignments of different augmentations (or “views”) of the same image, instead of comparing features directly as in contrastive learning. Grill et al.^15^ proposed a new approach to self-supervised image representation learning (BYOL) which relied on two neural networks, referred to as online and target networks, that interact and learn from each other. Chen et al.^16^ proposed SimSiam architecture to prove the simple Siamese networks can learn meaningful representations even without negative sample pairs, large batches and momentum encoders. Zbontar et al.^17^ proposed Barlow Twins by using an objective function that naturally avoids collapse by measuring the cross-correlation matrix between the outputs of two identical networks fed with distorted versions of a sample, and making it as close to the identity matrix as possible. Li et al.^18^ proposed a simple and effective self-supervised representation learning method based on the nearest minimum entropy of projected vectors.

Obviously, the setting in visual representation learning is very similar to the common scenario in underwater acoustic targets recognition. In this study, we mainly explore underwater acoustic target recognition with massive unlabeled samples and few labeled samples. First, taking the effect of data augmentation into account, empirical experiments are conducted on four typical underwater acoustic target recognition models by training models on different number of few labeled samples. Systematic performance baselines are drawn for subsequent experiments. Then, combining with advanced self-supervised learning frameworks and methods such as SimCLRv2^11^ and BYOL^15^, a learning framework containing four stages is proposed for training underwater acoustic target recognition model with a few labeled samples and massive unlabeled samples. The model is pre-trained on massive unlabeled samples through self-supervised learning firstly and then is fine-tuned on few labeled samples. Meanwhile, in order to alleviate the problem of limited performance during fine-tuning with very few labeled samples (1-shot, 5-shot or 20-shot), a semi-supervised fine-tuning method is proposed to improve the performance by mining and labeling partial unlabeled samples based on the similarity of deep features. Finally, unsupervised self-distillation is used to further improve the recognition performance of model. Furthermore, the joint model^19^ combining the wave and T-F representation-based models has been proved that can avoid the deviation caused by the limitation of a single model to a certain extent. In order to exploit the performance advantages of the joint model^19^ to achieve a satisfactory learning effect, the application scope of the framework is extended from the conventional single branch model to the two branches joint model. Datasets acquired from real-world scenarios are used to verify the effectiveness of the proposed framework and method. Using proposed learning framework and semi-supervised fine-tuning method improves the accuracy of four models. Especially for the joint model, the recognition accuracy has increased by 2.04% to 12.14% compared with the baselines. Only 10% of the labeled data is needed to exceed the model performance on the full dataset, effectively reducing the dependence of model on the number of labeled samples.

The main contributions of this study are summarized as follows:

- Systematic performance baselines are drawn on four typical underwater acoustic target recognition models by training models on datasets with different number of labeled samples. The extent of performance improvements can be accurately measured compared with the baselines.
- A learning framework containing four stages is proposed for training underwater acoustic target recognition model, which can make model achieve satisfactory performance with few labeled samples and massive unlabeled samples. The proposed learning framework is further extended to joint model with two branches for better learning effect.
- A semi-supervised fine-tuning method is proposed to improve the performance by mining and labeling partial unlabeled samples based on the similarity of deep features. The experimental results show that the proposed method improves the classification performance during fine-tuning with very few labeled samples.

The remainder of this report is organized as follows. The recent researches associated with this report are introduced and analyzed in the “Related work”. The learning framework with few labeled samples for underwater acoustic target recognition as well as the semi-supervised fine-tuning method are introduced in “Methods”. The dataset from real-world scenarios, data augmentation strategies and evaluation metrics are introduced in “Datasets and evaluations”. The experimental results and analysis are presented in “Results and discussions”. Conclusions and future works are presented in “Conclusion”.

## Related work

### Underwater acoustic target recognition

Inspired by self-supervised learning, several researches have explored self-supervised learning to solve the problem of labeled samples lacking in the underwater acoustic domain. Chen et al.^20^ taken Siamese networks to learn the difference between two inputs and made use of the difference between different categories to realize the stable tracking of the target on the multi-beam history graph by calculating the similarity between the target area and the search area. Liu et al.^21^ used a variety of data enhancement schemes, combined with transfer learning methods to achieve target classification. Liu et al.^22^ proposed a Siamese network to recognize the detection of envelope modulation on noise (DEMON) spectra of underwater target radiated noise and achieved good performance on simulated data. Wang et al.^23^ proposed the acoustic-embedding memory unit modified space autoencoder (ASAE) for self-supervised acoustic representation learning. The learned encoder module of ASAE will extract features which were used to train the shallow classification model for underwater target recognition task.

Despite the previous researches have been well done, there are some critical issues in underwater acoustic target recognition, compared to the studies in the field of image recognition. First, systematic performance baselines are lacking when learning with different number of few labeled samples, especially considering the performance gain brought by data augmentation^21^. It is necessary to confirm the extent of performance gains when training with different number of labeled samples. Second, in previous researches, either the dataset with massive unlabeled samples was not effectively utilized^20, 22^, or it has not been directly verified that using the unlabeled dataset can effectively alleviate the problem about the lack of labeled samples^23^. Besides, compared with few-shot learning methods, although the self-supervised learning methods get rid of the need for the base class dataset when solving few labeled samples problem, fine-tuning with very few labeled samples (1-shot or 5-shot) may have limited performance.

### Self-supervised learning

The most relevant self-supervised learning frameworks and methods to this report are SimCLRv2^11^ and BYOL^15^.

SimCLRv2^11^ is an effective self-supervised learning framework which consists three stages: self-supervised learning stage, supervised fine-tuning stage, and unsupervised self-distillation stage. The whole framework presents a progressive learning process. Through the self-supervised learning stage, the model is endowed with the primary perception ability to data, which is independent of specific downstream tasks and can be regarded as a kind of basic learning of context. Then, through supervised fine-tuning stage, the ability of the model is further extended and improved in specific recognition task scenarios, so that the model has the ability to distinguish and discriminate the types of underwater acoustic targets. Finally, through unsupervised self-distillation stage, the recognition performance of model is improved and generalized by massive unlabeled data. In such progressive learning, the self-supervised learning stage and unsupervised self-distillation stage improve the recognition performance by improving the initial weight and generalization of the model, hence the supervised fine-tuning stage is the key stage to introduce the recognition ability of specific targets. The model performance in this stage directly determines the model of the final recognition performance. However, supervised fine-tuning may not achieve the satisfactory results when the number of labeled sample is very small. The limited performance of supervised fine-tuning stage will become the bottleneck of the whole framework.

BYOL^15^ is a simple and effective self-supervised learning method. So far, BYOL^15^ has achieved comparable results to other self-supervised learning methods for its concise structure and no need of negative samples. There are two models in BYOL^15^. One is the online model, and the other is the target model. A data sample will be transformed twice by different degrees of data augmentation to obtain two different data views. Two data views are then input into the online model and the target model respectively for feature extraction. The extracted deep features are then embedded to a uniform vector space through projection networks. In the training process, the embedding vectors of online network are input into a prediction network to make the prediction of the embedding vectors of target model. The loss between the embedding vectors of target model and its predictions is used to update the parameters of online model. While the parameters of target model are updated by the exponential moving average of online model parameters. Many studies have shown that such gradient truncation can avoid model collapse and help self-supervised learning to achieve better results. Finally, the online model is saved as the learned representation pre-training model to provided initialization weight of models for the subsequent target recognition task.

## Methods

### Learning framework with few labeled samples

**Figure 1:**
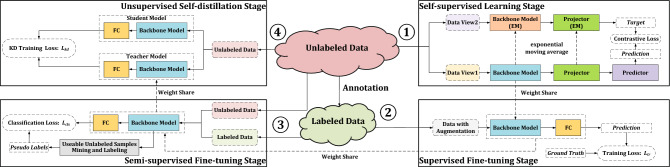
The learning framework with few labeled samples and massive unlabeled samples for underwater acoustic target recognition.

Combining advanced self-supervised learning frameworks SimCLRv2^11^ and BYOL^15^, a learning framework containing four stages is proposed for training underwater acoustic target recognition models, which can make model achieve satisfactory performance with few labeled samples and massive unlabeled samples, as shown in Fig. 1.

In self-supervised learning stage, massive unlabeled samples are used to train the backbone model through the contrastive learning schema which is widely used in representation learning and performs well. Then, in supervised fine-tuning stage, a fully connection (FC) layer is connected behind the trained backbone model, and few labeled samples are used to train them for classification task. When the number of labeled samples is very small (for example, only 1 or 5 labeled samples per class), the model trained in supervised fine-tuning stage may not perform well. It is difficult for the model to learn enough generalization to achieve the satisfactory results. In this case, after supervised fine-tuning stage, a semi-supervised fine-tuning stage will be executed to further improve model performance by mining and labeling partial unlabeled samples based on the similarity of deep features between labeled samples and unlabeled ones. Finally, unsupervised self-distillation is conducted where the fine-tuned model is used as the teacher model and a new randomly initialized model is used to fit the output of the teacher model on massive unlabeled samples.

Specifically, the proposed framework has two differences compared to related work. Firstly, the proposed framework includes four main stages: self-supervised learning stage, supervised fine-tuning stage, semi-supervised fine-tuning stage and unsupervised self-distillation stage. Different from SimCLRv2^11^, a semi-supervised fine-tuning stage is added between supervised fine-tuning stage and unsupervised self-distillation stage for fine-tuning with very few labeled samples (1-shot, 5-shot or 20-shot). Secondly, BYOL^15^ is used as the self-supervised learning method in self-supervised learning stage to improve the effect. In the proposed framework, the unlabeled samples are utilized more effectively to alleviate the problem without sufficient labeled sample.

#### Self-supervised learning stage

**Figure 2:**
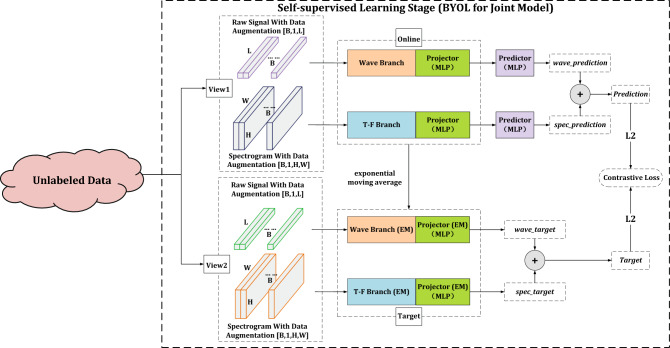
The details of using BYOL^15^ to conduct self-supervised learning on joint model^19^.

In self-supervised learning stage, advanced self-supervised learning method BYOL^15^ is used to pre-train model with unlabeled samples. BYOL^15^ can be directly used for self-supervised learning of conventional single branch deep convolutional neural network models. However, for the joint model^19^ which consists of wave and T-F branches, the model structure is different from the conventional models. It is necessary to adjust and modify the details so that the BYOL^15^ can be applied to the joint model^19^. Specifically, the details of using BYOL^15^ to conduct self-supervised learning on joint model are illustrated in Fig. 2.

For the wave branch and T-F branch of joint model, each branch has an independent projector network and a predictor network as well as the corresponding target projector network. The last fully connection layer of each branch is removed during self-supervised stage. Both projector and predictor networks are implemented by multi-layer perceptron (MLP). This MLP consists in a linear layer with output size 4096 followed by batch normalization^24^, rectified linear units (ReLU)^25^, and a final linear layer with output dimension 256. Due to the two-branch architecture of joint model, the final outputs of two branch lines are summed to obtain the predictions and targets in online and target networks respectively. The contrastive loss is defined as the mean squared error between the normalized predictions and targets.

After this stage of training, the backbone model will have effective perception capability for this kind of samples and is able to extract effective deep representations for input samples. However, since no labeling information is involved in the training process, the model cannot complete the specific classification task. Hence, in the next stage, the model will be supervised fine-tuned with labeled samples to recognize specific classes.

#### Supervised fine-tuning stage

In supervised fine-tuning stage, a fully connection (FC) layer is connected behind the trained backbone model to achieve the classification task. Few labeled samples are utilized to train both backbone model and FC layer.

In order to extend the proposed framework and exploit the performance advantages of the joint model^19^, in this report, the synchronous deep mutual learning method^19^ is used to train the joint model during supervised fine-tuning stage. Specifically, let $\Theta _{wave}$ and $\Theta _{T-F}$ denote the wave and T-F branches of the joint model, respectively. The probability distribution of final prediction result is denoted as $p_{joint}$. In the training process, the probability distributions of the T-F and wave branch transformed by soft-max layers are denoted as $p_{wave}$ and $p_{T-F}$, respectively. For a batch of samples *x* and the corresponding labels $y_{x}$, the mutual learning loss is defined as:

1 $$\begin{aligned} \mathscr {L}{L}_{ml}(\Theta _{wave},\Theta _{T-F}) = \mathscr {L}{L}_{MSE}(p_{wave},p_{T-F}) \end{aligned}$$

where $\mathscr {L}{L}_{MSE}(p_{wave},p_{T-F})$ denotes the mean squared error (MSE) loss between $p_{wave}$ and $p_{T-F}$. Meanwhile, the standard cross-entropy loss $\mathscr {L}{L}_{CE}$ is also used to minimize the diversity between $p_{joint}$ and ground truth $y_{x}$. Hence, the unified joint loss function for training joint model is defined as:

2 $$\begin{aligned} \mathscr {L}{L}_{joint}(\Theta _{wave},\Theta _{T-F}) = \mathscr {L}{L}_{CE}(p_{joint}/2,y_{x}) + \mathscr {L}{L}_{ml}(\Theta _{wave},\Theta _{T-F}) \end{aligned}$$

After this stage of training, the model will have a certain identification ability on the specific classification task. However, limited by the small number of labeled samples, fine-tuning training may face few samples or even 1, 5 or 20 samples per class. Therefore, the semi-supervised fine-tuning will be executed to further improve model performance.

#### Semi-supervised fine-tuning stage

In semi-supervised fine-tuning stage, both labeled and unlabeled samples are used in training process. The unlabeled samples are mainly used to increase the generalization of the model. In other words, let the model learn more samples under the classification task. There are two key points. One is how to assign class information to these unlabeled samples. Another is how to avoid excessive label noise to affect model convergence speed and accuracy. Because manual labeling is not considered, the label noise is almost inevitable such as incorrect or unstable label assignment for an unlabeled sample. In this study, a semi-supervised fine-tuning method is proposed to improve the fine-tuning performance by mining and labeling partial unlabeled samples based on the similarity of deep features between labeled samples and unlabeled samples. In addition, based on the structure of the joint model, a consistent matching module is designed in the proposed semi-supervised fine-tuning method to reduce label noise by using the consistency principle between branch models. The details of proposed method will be described in Section Semi-supervised fine-tuning method.

Meanwhile, the labeled samples also participate in training process. The labeled samples will not only be used to assign labels to unlabeled samples, but also be used to calculate classification losses to ensure that the performance of model is not worse than the fine-tuned model in previous stage.

#### Unsupervised self-distillation stage

Only unlabeled samples are used in this stage. The fine-tuned model is used as the teacher model and a new randomly initialized model is used to fit the output of the teacher model on massive unlabeled samples. This stage is similar to SimCLRv2^11^ in which the unsupervised self-distillation^26^ has been shown to further improve the performance of fine-tuning models.

**Figure 3:**
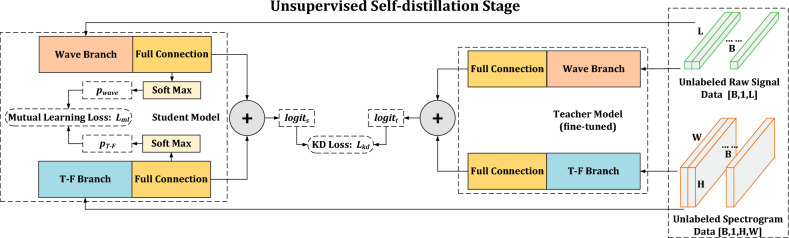
The details of unsupervised self-distillation stage on joint model^19^.

Similar to other stage, in order to extend the proposed framework and exploit the performance advantages of the joint model^19^, mutual learning loss is used for joint model in the training of student networks, because the mutual learning loss is calculated regardless of category. The details of unsupervised self-distillation stage on joint model are illustrated in Fig. 3.

The output of teacher model is denoted as $logit_{t}$, which is the sum of wave and T-F branch after their full connection layer. Correspondingly, the output of student model is denoted as $logit_{s}$. The distillation loss is defined as:

3 $$\begin{aligned} \mathscr {L}{L}_{kd}= \mathscr {L}{L}_{CE}\left(\sigma \left(\frac{logit_{s}}{T}\right),\sigma \left(\frac{logit_{t}}{T}\right)\right) \end{aligned}$$

where $\mathscr {L}{L}_{CE}(.,.)$ denotes the cross-entropy loss, $\sigma (.)$ denotes the soft-max function, and *T* is a temperature hyperparameter. Meanwhile, the mutual learning loss is also used to minimize the diversity between $p_{wave}$ and $p_{T-F}$. Hence, the unsupervised self-distillation loss function for training joint model is defined as:

4 $$\begin{aligned} \mathscr {L}{L}_{ukd}= \mathscr {L}{L}_{kd} + \mathscr {L}{L}_{ml}(\Theta _{wave},\Theta _{T-F}) \end{aligned}$$

### Semi-supervised fine-tuning method

**Figure 4:**
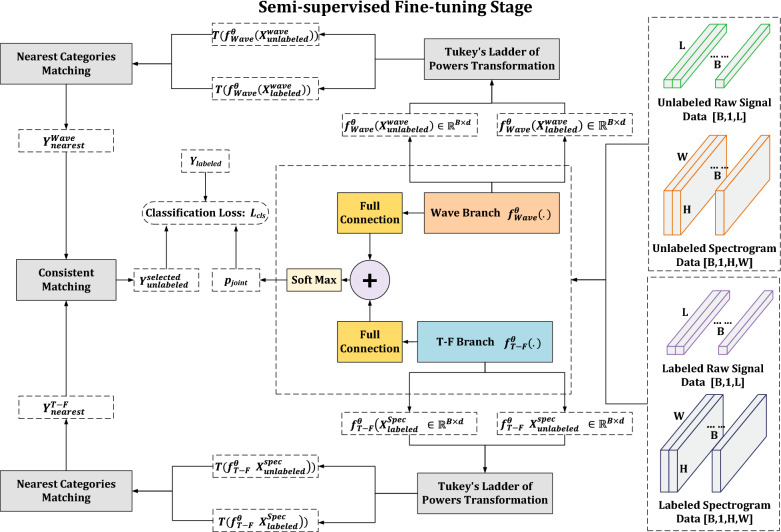
The semi-supervised fine-tuning method.

The semi-supervised fine-tuning method proposed in this study is shown in Fig. 4. To avoid tautology, the presentation of semi-supervised fine-tuning method is directly based on the joint model^19^. The case applied to conventional single branch deep neural networks is easily obtained based on it.

At the beginning, a batch of unlabeled and labeled samples are fed to joint model for the forward calculation. Then, the outputs of two branches’ backbone networks are extracted as the deep features of unlabeled and labeled samples. Based on the extracted deep features, three important modules will be used to mining and labeling partial unlabeled samples, which are: the Tukey’s ladder of powers transformation module, the nearest categories matching module and the consistent matching module. As mentioned before, there are two key points in semi-supervised fine-tuning stage: how to assign class information to these unlabeled samples, and how to avoid excessive label noise to affect model training. In the proposed method, the Tukey’s ladder of powers transformation module is taken to reduce the distribution deviation of deep features and ensure the stability of the subsequent calculation to a certain extent. The nearest categories matching module is designed to assign labels to unlabeled samples as correct as possible through the similarity between deep features of labeled and unlabeled samples. The consistent matching module is used further reduce label noise and retain as many correctly labeled samples as possible. For conventional single branch deep neural networks, only a unique pseudo-label is obtained in the nearest categories matching module. In this case, the consistent matching module is no longer needed.

After the processing of three modules, a part of unlabeled samples will be assigned category labels. The unlabeled samples assigned category labels are used together with the labeled samples to calculate classification loss by comparing their labels with the predicted outputs of the model.

Assume the wave branch excluding the full connection layer of the joint model is denoted as $f_{Wave}^{\Theta }(.)$ and the T-F branch excluding the full connection layer of the joint model is denoted as $f_{T-F}^{\Theta }(.)$. A batch of unlabeled samples are denoted as $X_{unlabeled}^{wave}$ and $X_{unlabeled}^{spec}$ corresponding to their waveform and T-F representations respectively. Similarly, a batch of labeled samples are denoted as $X_{labeled}^{wave}$ and $X_{labeled}^{spec}$, as well as their labels $Y_{labeled}$.

#### Tukey’s ladder of powers transformation module

The Tukey’s ladder of powers transformation has proved to be effective in reducing the deviation of the deep features distributions in some few-shot learning methods^27–29^. It makes the deep features of different samples or different datasets comparable and helps stabilizing the training process.

First, a batch of unlabeled and labeled samples are input into joint model to obtain the corresponding deep features $f_{Wave}^{\Theta }(X_{unlabeled}^{wave})$, $f_{Wave}^{\Theta }(X_{labeled}^{wave})$, $f_{T-F}^{\Theta }(X_{unlabeled}^{spec})$, and $f_{T-F}^{\Theta }(X_{labeled}^{spec})$. Then, the Tukey’s ladder of powers transformation is adopted to reduce the deviation of the distribution. The transformation makes the distribution of deep features close to a Gaussian distribution, which is convenient for subsequent matching. Specifically, if *x* is an input deep feature vector, the function of Tukey’s ladder of powers transformation can be expressed as:

5 $$\begin{aligned} T(x) = \left\{ {\begin{array}{*{20}{l}} x^\lambda &{}\lambda \ne 0\\ log(x)&{}\lambda = 0 \end{array}} \right. , \end{aligned}$$

where *T*(*x*) is the feature vector after transformation, and $\lambda$ is a hyperparameter to adjust the mapping distribution. Specially, the transformed deep features of unlabeled and labeled samples can be denoted as $T(f_{Wave}^{\Theta }(X_{unlabeled}^{wave}))$, $T(f_{Wave}^{\Theta }(X_{labeled}^{wave}))$, $T(f_{T-F}^{\Theta }(X_{unlabeled}^{spec}))$, and $T(f_{T-F}^{\Theta }(X_{labeled}^{spec}))$.

#### Nearest categories matching module

In the next, the nearest categories matching is used to find the close category for each unlabeled sample. For each branch of joint model, there are two sets of deep features corresponding to labeled and unlabeled samples. The distances between the transformed deep features of two sets will be calculated first. Specifically, cosine similarity is considered as the distance measurement in this study. The distances between deep features of labeled and unlabeled samples can be calculated by:

6 $$\begin{aligned} disWave=\left\{ \frac{(T(f_{Wave}^{\Theta }(x_{unlabeled}^{wave})))^T \cdot T(f_{Wave}^{\Theta }(x_{labeled}^{wave}))}{\Vert T(f_{Wave}^{\Theta }(x_{unlabeled}^{wave}))\Vert \cdot \Vert T(f_{Wave}^{\Theta }(x_{labeled}^{wave}))\Vert }, \quad \forall x_{unlabeled}^{wave} \in X_{unlabeled}^{wave} \quad and \quad \forall x_{labeled}^{wave} \in X_{labeled}^{wave} \right\}, \end{aligned}$$

and

7 $$\begin{aligned} disSpec=\left\{ \frac{(T(f_{T-F}^{\Theta }(x_{unlabeled}^{spec})))^T \cdot T(f_{T-F}^{\Theta }(x_{labeled}^{spec}))}{\Vert T(f_{T-F}^{\Theta }(x_{unlabeled}^{spec}))\Vert \cdot \Vert T(f_{T-F}^{\Theta }(x_{labeled}^{spec}))\Vert }, \quad \forall x_{unlabeled}^{spec} \in X_{unlabeled}^{spec} \quad and \quad \forall x_{labeled}^{spec} \in X_{labeled}^{spec} \right\}, \end{aligned}$$

where *disWave* and *disSpec* represent the transformed feature distances of wave and T-F branches. For an unlabeled sample, define the distance from it to a category as the average distance of deep features between the sample and all labeled samples which belong to the same category:

8 $$\begin{aligned} disC_{wave}(x_{unlabeled}^{wave},y_{i})=mean \{ disWave(x_{unlabeled}^{wave}, x_{labeled}^{wave})\}, \quad \forall x_{labeled}^{wave} \in X_{labeled}^{wave}\ and\ lable\ of\ x_{labeled}^{wave}=y_{i} \end{aligned}$$

and

9 $$\begin{aligned} disC_{spec}(x_{unlabeled}^{spec},y_{i})=mean \{ disSpec(x_{unlabeled}^{spec}, x_{labeled}^{spec})\}, \quad \forall x_{labeled}^{spec} \in X_{labeled}^{spec}\ and\ lable\ of\ x_{labeled}^{spec}=y_{i} \end{aligned}$$

where $y_{i} \in Y_{labeled}$. Then the category which is the closest to the unlabeled sample in average distance will be used as a pseudo-label of unlabeled sample. In the end, each unlabeled sample will get two pseudo-labels $y_{nearest}^{wave}$ and $y_{nearest}^{T-F}$ from wave and T-F branches respectively.

#### Consistent matching module

Because manual labeling is not considered, the noise of pseudo-label is almost inevitable such as incorrect or unstable label assignment for an unlabeled sample. Hence, the consistent matching module will be used to mitigate the label noise and give the uniform assignment. Specifically, a consistency condition is defined such that an unlabeled sample will be selected for computing the classification loss only if its $y_{nearest}^{wave}$ is equal to $y_{nearest}^{T-F}$.

The reason for setting this condition is that the unlabeled samples of which $y_{nearest}^{wave}$ is not equal to $y_{nearest}^{T-F}$ are considered unstable for training. The feature representations from the two branches diverge and cannot reach agreement. The unlabeled samples need unique labels for calculating the loss, therefore the samples that do not meet the condition will not have an impact on updating of model weight. The noise of pseudo-labels can be effectively reduced.

## Datasets and evaluations

In this section, The dataset from real-world scenarios, data augmentation strategies and evaluation metrics are introduced.

### Datasets

DeepShip dataset^30^ being acquired form real-world scenarios is used to evaluate models in this study. DeepShip is an underwater acoustic benchmark dataset proposed in recent years. It consists of 47 h and 4 min of real-world underwater recordings of 265 different ships belonging to four categories. The data source is from Ocean Networks Canada. Specifically, we construct the dataset through the same strategy used in^19^, where the background noise data supplemented from the same data source and each recording is sliced into segments of 3 s. The division of the training and test datasets also remain consistent and segments from one recording cannot be concurrently split into training and testing datasets.

Differently, to conduct the empirical experiments with different number of few labeled samples, the labeled dataset is constructed by randomly sampling training dataset according to a certain proportion. Overall, there are seven different training datasets, each containing a different number of samples. Specifically, one of them is the full training dataset (100%), three of them are the few labeled training dataset (10%, 5% and 1%), and three of them are training datasets with extremely small number of labeled samples (20-shot, 5-shot, 1-shot). There is only one testing dataset and no overlap with each of the training datasets. The number of samples in all training and testing datasets is shown in Table 1.

**Table 1:** Number of samples in all training and testing datasets. About 10%, 5%, and 1% samples per class are randomly sampled in training dataset to construct few labeled datasets. 20, 5, and 1 samples per class are randomly sampled in training dataset to construct extremely few labeled datasets.

Class label	Tanker	Cargo	Tug	Passenger ship	BG
Total number of samples	14,924	12,865	13,535	15,540	13,800
Number of training samples (100%)	11,123	9066	9741	11,734	10,000
Number of training samples (10%)	1112	906	974	1173	1000
Number of training samples (5%)	556	453	487	586	500
Number of training samples (1%)	111	90	97	117	100
Number of training samples (20-shot)	20	20	20	20	20
Number of training samples (5-shot)	5	5	5	5	5
Number of training samples (1-shot)	1	1	1	1	1
Number of testing samples	3801	3799	3794	3806	3800

Different sizes of training datasets can provide a clear performance baseline, which can be used to systematically evaluate the recognition performance of the model with few labeled samples.

### Data augmentation and evaluations

When training with few labeled samples, using data augmentation may improve the model recognition performance. Besides, data augmentation will be used in self-supervised learning stage to generate different data views. Data augmentation is unavoidable in the whole learning framework. Therefore, it is necessary to consider the impact of data augmentation when constructing the baseline.

**Figure 5:**
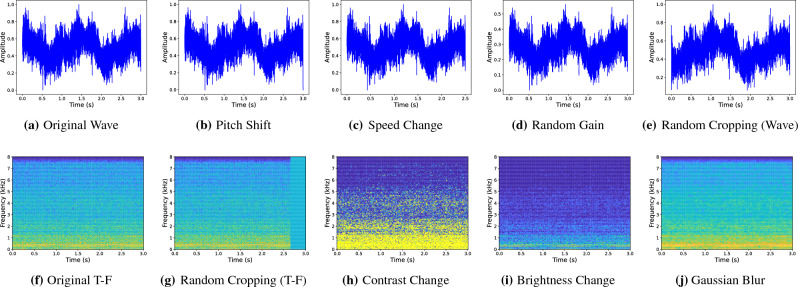
Illustrations of data augmentation.

Concretely, for the two data representations of joint model, different data augmentation strategies are adopted inspired by^21^. For waveform representation, there are four data augmentation methods used for training as shown in Fig. 5a–e:

- Pitch shift: randomly shift the pitch of waveform based on the uniform distribution.
- Speed change: randomly change the audio speed of waveform based on the uniform distribution.
- Random gain: amplify or decrease the audio signal of waveform randomly.
- Random cropping: randomly crop the waveform into a fixed-length along the time axis.

For T-F representation, there are four data augmentation methods used for training as shown in Fig. 5f–j:

- Random cropping: randomly crop the spectrogram into a fixed size and then resize back to the original size.
- Contrast change: randomly change the contrast of spectrogram based on the uniform distribution.
- Brightness change: randomly change the brightness of spectrogram based on the uniform distribution.
- Gaussian blur: randomly add Gaussian noise into spectrogram based on the uniform distribution.

All data augmentation strategies are applied to the input samples with a certain probability during training stage.

The classification performances of models in this study are evaluated by accuracy which is defined as the ratio of correct identifications.

10 $$\begin{aligned} Accuracy=\frac{Num\_correct}{Num\_all} \times 100\% \end{aligned}$$

where $Num\_correct$ represents the number of samples predicted by model correctly. $Num\_all$ represents the total number of samples participating in the test.

All experiments are conducted on a regular rack server with four Nvidia Titan RTX GPU (24G). Training and testing models are both based on the GPU. All neural networks are implemented on the open source machine learning framework pytorch-1.6.0 under Linux operating system with python programming language.

## Results and discussions

The experiments are designed around three aspects. First, empirical experiments which take the effect of data augmentation into account are conducted. The systematic performance baselines are drawn on four typical underwater acoustic target recognition models by training models on seven training datasets. Second, comparative experiments are conducted in which the recognition performance is used to evaluate the proposed learning framework. Third, ablation experimental results of some stages are used to evaluate the proposed semi-supervised fine-tuning method.

### Empirical experiments for baselines

In order to build a reliable baseline, four typical underwater acoustic target recognition models are trained on seven training datasets including MLENET^31^, lightweight MSRDN^19^, SCAE^30^ and joint model^19^. Both MLENET^31^ and SCAE^30^ are T-F representation-based models which achieved good results in underwater acoustic target recognition. The lightweight MSRDN^19^ is an efficient wave-based model for underwater acoustic target recognition. The joint model^19^ used in this report takes lightweight MSRDN^19^ as the wave branch and takes the encoder part of SCAE^30^ as the T-F branch. Because the computations of two standard convolution layers at the beginning of the SCAE^30^ encoder cause a large number of FLOPs, the second standard convolution layer is replaced by a max pooling layer. The modified model has less computation and its performance will not be greatly affected, which is beneficial to the training experiment of the joint model.

**Figure 6:**
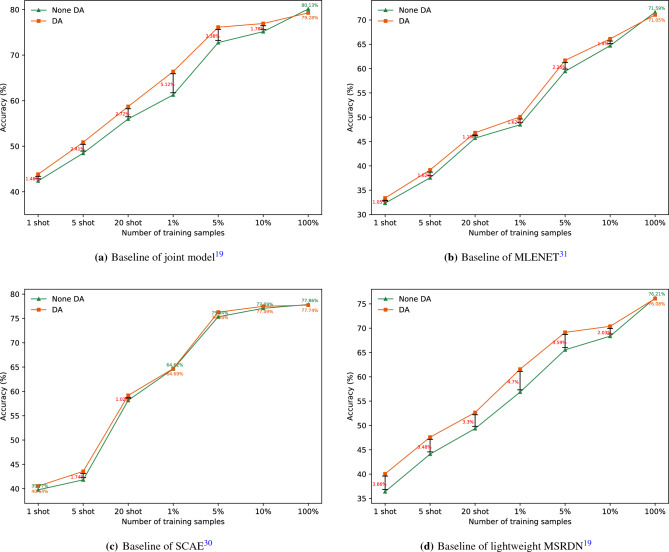
Baselines of the four models on seven training datasets.

We trained all networks from scratch and used AdamW optimizer^32^ with 0.05 weight decay. The batch size was set to 64 for each GPU in all experiments. The learning rate ranges from 1e−3 to 1e−8, and varies following a cosine annealing schedule. We regularized all networks using label smoothing^33^ with 0.1 epsilon on entropy loss. The best model was saved and the testing set was evaluated during the optimization process. The entire training process ran through the training set approximately 220 times (220 epochs). Each model was then fully trained. The experimental results of four models on seven training datasets are presented in Fig. 6.

From experimental results, we can find out that the performances of the models are closely related to the number of training examples. As the number of samples decreases, the performances of four models shows a significant decrease. Data augmentation can improve model performance during training with small sample size, but the effect of improvement varies from model to model. The accuracy of joint model is increased by 1.78% to 5.12%. The accuracy of MLENET^31^ is increased by 1.05% to 2.25%. The accuracy of SCAE^30^ is increased by 0.07% to 1.74%, and that of Lightweight MSRDN^19^ is increased by 2.03% to 4.70%. We observe that when training on 100% training dataset, employing data augmentation hurts the performance of model. This phenomenon is related to our strong data augmentation strategies and is consistent with the experimental results in^10^. It reflects that the appropriate strength of data augmentation is different in the face of different number of training samples.

The experimental results proves that it is not enough to rely on data augmentation alone. On the one hand, the effect of data augmentation is limited. On the other hand, for different models and training datasets, it may be necessary to try different data augmentation intensification several times to find the best applicable strategy. Therefore, new learning framework need to be studied to solve the problem of performance drop during training with few labeled samples.

### Comparative experiments

Four typical underwater acoustic target recognition models are trained under the proposed learning framework. Specifically, in self-supervised learning stage, all samples are treated as unlabeled data, and the label information will not be used in this stage. Then, in supervised fine-tuning stage, the model is first loaded with the weights trained in the previous stage and then fine-tunes on different numbers of samples. The training parameters of the supervised fine-tuning stage are consistent with the baseline experiment. After supervised fine-tuning stage, models trained in the extreme few-shot case (1-shot, 5-shot, and 20-shot) will continue to be trained through the semi-supervised fine-tuning stage. Finally, all models will be trained by unsupervised self-distillation on all samples without labels. The entire training process ensures that the model has only seen partial label information determined by corresponding training datasets. Compared with the performance baselines of four models on seven training datasets, the improved accuracy of the models after training with the proposed learning framework is presented in Table 2, in which the values represent the improvements (or decreases) of accuracy index compared to the best performance of baseline. A positive value represents a further improvement in model accuracy when using the learning framework compared to the best performance baseline. Negative values mean that the accuracy decreases after the learning framework is used.

**Table 2:** Improved accuracy of four models on seven training datasets compared with the baselines.

Datasets	1-shot (%)	5-shot (%)	20-shot (%)	1% (%)	5% (%)	10% (%)	100% (%)
MLENET^31^	7.00	0.95	7.00	9.13	3.74	2.31	0.46
Lightweight MSRDN^19^	6.02	5.64	7.40	8.11	4.60	3.94	1.11
SCAE^30^	8.12	5.49	1.26	0.94	0.91	1.25	$-0.02$
Joint model^19^	2.04	6.76	5.74	7.02	3.19	3.22	0.82

It can be seen from the experimental results that the recognition accuracy of the model has been further improved after using the proposed learning framework with few labeled samples. On the 1-shot dataset, the accuracy of four models improved by 2.04% to 8.12%. On the 5-shot dataset, the accuracy of four models increased by 0.95% to 6.76%. On the 20-shot dataset, the accuracy of four models increased by 1.26% to 7.40%. On few labeled sample datasets of 1% to 10%, the accuracy of four models has also improved by 0.91% to 9.13%.

On the full training data set (100%), except that the accuracy of SCAE^30^ slightly decreased by 0.02% compared with the baseline with no data augmentation, the recognition accuracy of the remaining models even increased by 0.46% to 1.11%. The reason of this phenomenon may be that the pre-training through self-supervised learning improves the initial weights of the model. The experimental results also proves that the learning framework proposed in this report can solve the problem that the use of strong data augmentation strategies on full training dataset will damage the model recognition effect to a certain extent. Taking the joint model as a specific example, Table 3 shows the comparison with baseline performance. Baselines+DA row and Baselines row represent the accuracy of the joint model on the seven datasets with or without data augmentation respectively.

**Table 3:** Comparative experimental results of joint model on seven training datasets.

Datasets	1-shot (%)	5-shot (%)	20-shot (%)	1% (%)	5% (%)	10% (%)	100% (%)
Baselines	42.38	48.46	55.99	61.27	72.75	75.16	**80.13**
Baselines+DA	**43.84**	**50.87**	**58.71**	**66.39**	**76.13**	**76.94**	79.27
Ours	45.88 (+2.04)	57.63 (+6.76)	64.45 (+5.74)	73.41 (+7.02)	79.32 (+3.19)	80.16 (+3.22)	80.95 (+0.82)

The bold values represent the best performances of baselines on each training datasets. The numbers in parentheses represent the improvement over the best baselines on each training datasets.

Compared with baselines with data augmentation, the performance of joint model has been improved through the training of proposed learning framework. On few labeled datasets (10%, 5%, 1%) and extremely few labeled datasets (20-shot, 5-shot, 1-shot), the recognition accuracy of model is improved by 2.04% to 7.02%. On full (100%) training dataset, the recognition accuracy of model is improved by 1.68%. The problem that strong data augmentation can hurt model performance on full training dataset is solved by using the proposed learning framework. Moreover, on the 5% training dataset, the model achieves better accuracy than the model supervised trained on full (100%) training dataset with data augmentation.

Compared with the baselines without data augmentation, the improvements of accuracy are even more significant on few labeled datasets (10%, 5%, 1%) and extremely few labeled datasets (20-shot, 5-shot, 1-shot). The recognition accuracy of model is improved by 3.5% to 12.14% from different few labeled datasets. On full (100%) training dataset, the recognition accuracy of model is also improved by a small margin (0.82%). Specifically, on the 10% training dataset, the model narrowly exceeds (0.03%) the accuracy of supervised training on full (100%) training dataset. It shows that only 10% of the training dataset is needed to be labeled to achieve the best performance of the model.

**Figure 7:**
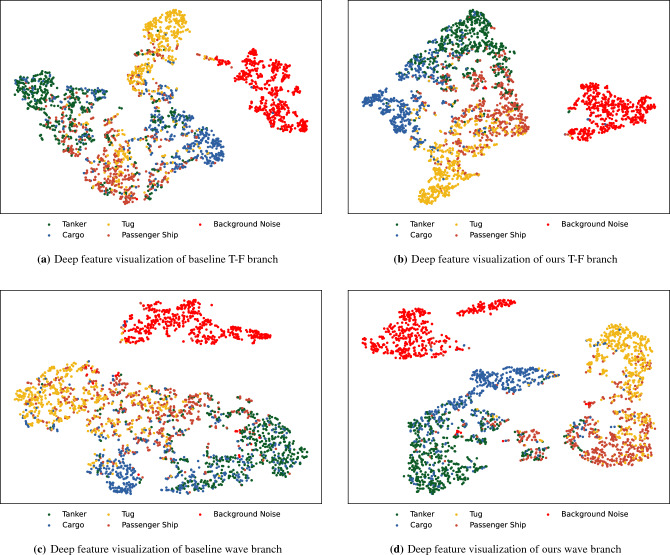
t-SNE visualization of output feature vectors of the joint model branches on 5% training dataset.

In addition, in order to show the improvement of model recognition performance with few labeled samples, feature visualization method t-distributed stochastic neighbor embedding (t-SNE)^34^ is used to observe the separability of deep features. 500 samples for every class are selected randomly from test datasets. The joint model is trained on 5% training dataset. Outputs of the backbone network (before full connected layer) of two branches of joint model are extracted as learned deep features which are visualized in Fig. 7. Through the visual comparison of deep features, it can be seen that after using the proposed learning framework, the features output by the backbone of model are more category separable.

The experimental results demonstrate that the learning framework proposed in this study can effectively improve the performance of the model with different small number of labeled samples. The dependence of the model on the number of labeled samples is greatly reduced. Besides, the learning framework proposed in this study also improve the performance of the model with full training samples. It proves that the proposed learning framework can learn information beyond labels, such as the essential characteristics and representations of underwater acoustic data.

### Ablation experiments

To evaluate semi-supervised fine-tuning method and clarify the performance gain of each stage, the ablation experiments are conducted on the extreme few-shot datasets (1-shot, 5-shot, and-20 shot). For the convenience of description, the four stages in the learning framework are denoted as S1, S2, S3, and S4 according to their execution order. On each dataset, the model performance in five cases is used for comparison:

- Baseline + DA: the best performances of baselines on each training datasets.
- S1 + S2: only use self-supervised learning stage and supervised fine-tuning stage to train the model.
- S1 + S2 + S3: after self-supervised learning stage and supervised fine-tuning stage, train the model by proposed semi-supervised fine-tuning method.
- S1 + S2 + S4: after self-supervised learning stage and supervised fine-tuning stage, directly train the model in unsupervised self-distillation stage.
- S1 + S2 + S3 + S4: the full stages of the proposed learning framework.

“S1 + S2” represents using BYOL^15^ for pre-training and using mutual learning^19^ for fine-tuning. “S1 + S2 + S4” represents using SimCLRv2^11^ as learning framework in which the self-supervised method is implemented by BYOL^15^. “S1 + S2 + S3” represents an intermediate variant of the proposed learning framework. Such a set of controlled experiments can clearly show the points of performance gains of the proposed learning framework and demonstrate the performance of the proposed semi-supervised fine-tuning method.

**Figure 8:**
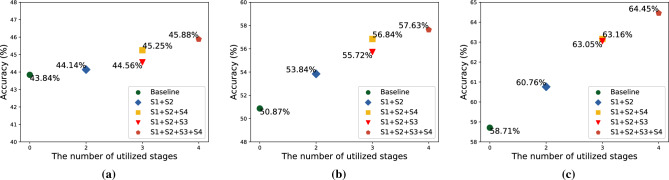
Comparison of the joint model performance under five cases on three extreme few-shot datasets. (**a**) Results on 1 shot dataset; (**b**) results on 5 shot dataset; (**c**) results on 20-shot dataset. The X-axis coordinate represents the number of stages utilized in training process, and the Y-axis represents the accuracy of the joint model. Each shape point represents a stage combination. The number near the shape points represents the accuracy of the joint model.

The experimental results for ablation experiments are illustrated in Fig. 8. The experimental results show that the learning framework proposed in this study can obtain the maximum performance gain. Compared with the accuracy of model in “S1 + S2” case, the accuracy of model in “S1 + S2 + S3” case improves 2.31%, 1.88%, and 2.29% on 1-shot , 5-shot , and 20-shot datasets respectively. The accuracy of model in “S1 + S2 + S4” case improves 3.00%, 3.00%, and 2.40% on 1-shot, 5-shot, and 20-shot datasets respectively. This phenomenon demonstrates that after the supervised fine-tuning stage, both self-distillation and semi-supervised learning are able to further improve the performance of the model. The possible reason is that both stages utilize unsupervised datasets and are able to extend the generalization of the model with unlabeled samples.

In contrast, the proposed semi-supervised fine-tuning method is able to bring higher performance gains than unsupervised self-distillation. The recognition performance of the joint model can be further improved when all stages are used. It proves that the proposed semi-supervised fine-tuning method can improve the training effect of the model under the condition of extreme few labeled samples.

### Discussions and analysis

The learning framework proposed in this study can effectively improve the model performance with few labeled samples and massive unlabeled samples. We believe that the reason for the improvement is the full utilization of a large number of unlabeled samples. In fact, there are three stages where unlabeled samples are used in the learning framework. In self-supervised learning stage, the unlabeled samples are used to give model the perception ability for underwater acoustic data. This class-independent perception is actually reflected in the more suitable model weights than randomly initialized. In semi-supervised fine-tuning stage and unsupervised self-distillation stage, the unlabeled samples are treated as a source of auxiliary information. Information beyond the range of labeled samples is used to improve the generalization of model.

However, despite the improvement, the performances of model are still not satisfactory in the case of extremely few labeled samples. We think that the reason is excessive noise of the extra information introduced from the unlabeled datasets. In semi-supervised fine-tuning stage, the smaller labeled samples, the worse the performance of the fine-tuned model of supervised fine-tuning stage, and the worse the similarity of the feature expression of the samples in the same class. Hence, the label noise of assigned unlabeled samples is inevitably increase. In unsupervised self-distillation stage, the situation is similar. A poorly performing teacher network is bound to limit the performance of the student network. In fact, such phenomenon is consistent with no free lunch theorem. In the absence of sufficient information to constrain the learning, it is difficult for the model to achieve satisfactory performance. The learning framework and semi-supervised fine-tuning method proposed in this study are able to increase the constraints of model learning to some extent. The dependence of model on labeled samples is greatly reduced, but it is still impossible to completely get rid of the constraints of labeled samples.

Besides, as a multistage learning framework, the duration of the whole training process is longer than that of classical supervised training. In the experiments of this report, it may take 4-5 days to complete the training of a model and it does not change the inference delay of the models. However, the time cost of proposed learning framework is almost negligible compared to the time cost of manually annotating a large amount of data. The time required to label a full dataset is measured on a monthly basis. By using proposed learning framework, a lot of time cost of annotation is saved and the trouble of dataset labeling error is also alleviated. For example, the joint model only needs 10% of full labeled dataset to achieve the same or even better results.

## Conclusion

Underwater acoustic target recognition under the condition of massive unlabeled samples and few labeled samples is explored in this study and a learning framework with four stages is proposed following advanced self-supervised learning frameworks such as SimCLRv2^11^ and BYOL^15^. Meanwhile, in order to alleviate the problem of limited performance during fine-tuning with very few labeled samples (1-shot or 5-shot), a semi-supervised fine-tuning method is proposed to improve the performance by mining and labeling partial unlabeled samples based on the similarity of deep features. Using proposed learning framework, only 10% of the training dataset is needed to be labeled to achieve the best performance of the joint model. Compared to the consecutive performance baselines, the performances of four models are all improved on datasets with different number of labeled samples. Besides, the ablation experiments have demonstrated the effectiveness of proposed semi-supervised fine-tuning method.

In the future study, we will carry out research on how to use unlabeled samples to generate more constraint information for model training and reduce the dependence on labeled samples as much as possible to further improve the performance when training with extremely few labeled samples.

## Data Availability

The data source used in this study were provided by Ocean Networks Canada. All the source data can be downloaded at: https://www.oceannetworks.ca/. However, in this study, we actually use the DeepShip dataset^30^ which is produced based on the source data of Ocean Networks Canada. This dataset is actually an open source dataset and can obtain by contacting Dr. Shengzhao Tian via shengzhao_tian@163.com.
